# Optimization of skin dose using *in‐vivo* MOSFET dose measurements in bolus/non‐bolus fraction ratio: A VMAT and a 3DCRT study

**DOI:** 10.1002/acm2.12525

**Published:** 2019-01-09

**Authors:** Anabela G. Dias, Diana F. S. Pinto, Maria F. Borges, Maria H. Pereira, João A. M. Santos, Luís T. Cunha, Joana Lencart

**Affiliations:** ^1^ Medical Physics Department Portuguese Institute of Oncology (IPO‐Porto) Porto Portugal; ^2^ Medical Physics Radiobiology and Radiation Protection Group Research Centre, Portuguese Institute of Oncology Porto (CI‐IPO) Portugal; ^3^ Radiotherapy Department Portuguese Institute of Oncology Porto Portugal; ^4^ Abel Salazar Institute of Biomedical Sciences (ICBAS) University of Porto Porto Portugal

**Keywords:** bolus, breast carcinoma, *in‐vivo* dosimetry, MOSFETs, skin dose

## Abstract

*In‐phantom* and *in‐vivo* three dimensional conformal radiation therapy (3DCRT) and volumetric modulated arc therapy (VMAT) skin doses, measured with and without bolus in a female anthropomorphic phantom RANDO and in patients, were compared against treatment planning system calculated values. A thorough characterization of the metal oxide semiconductor field effect transistor measurement system was performed prior to the measurements in phantoms and patients. Patients with clinical indication for postoperative external radiotherapy were selected. Skin dose showed higher values with 3DCRT technique compared with VMAT. The increase in skin dose due to the use of bolus was quantified. It was observed that, in the case of VMAT, the bolus effect on the skin dose was considerable when compared with 3DCRT. From the point of view of treatment time, bolus cost, and positioning reproducibility, the use of bolus in these situations can be optimized.

## 1 INTRODUCTION

Skin injury is a known consequence of breast external radiotherapy treatments. These effects occur particularly in areas subjected to friction such as axilla and skin folds.^1^, ^2^ The severity of these radiation‐induced side effects depends on several factors such as radiotherapy treatment modality and planning details; for instance fractionation, total dose, use of bolus, field modifiers, site, concurrent chemotherapy, and the use of biological agents.^1^, ^2^, ^3^, ^4^, ^5^, ^6^, ^7^ Skin reactions become more evident toward the end of the treatment with their highest severity generally occurring in the first 2 weeks after the end of the treatment.^1^, ^2^, ^3^, ^4^, ^8^

The skin is generally considered an organ at risk and the minimization of the skin dose is a concern after breast conservative and mastectomy postoperative irradiation. However, in some cases, as inflammatory breast cancer or locally advanced breast cancer T4, the delivery of therapeutic dose to the skin is clinically beneficial. In this situation it is necessary to calculate and measure the superficial dose.^3^, ^4^, ^10^, ^11^, ^12^ The precision of skin dose assessment is essential to guarantee that on one hand, the dose is below the tolerance levels and, on the other hand, required doses should be high enough to avoid tumor recurrence.^3^, ^4^, ^6^, ^10^, ^11^, ^12^ Dose prescription and dosimetry assessment in the skin is not a straightforward task due to the limitation of treatment planning system (TPS) when calculating dose at the surface and at different structures of the skin (basal and dermal layers), as depth and location vary between patients and even in the same patient. The majority of available dose calculation algorithms are not sufficiently accurate to compute dose distributions in superficial and build‐up regions where electron equilibrium is not established. Generally, TPS can calculate skin dose (build‐up region) with accuracy up to 20%.^11^, ^12^, ^13^, ^14^, ^15^

Different methodologies for skin dose measurement and detectors characterization have been reported in the literature: radiochromic films; thermoluminescent dosimeters (TLD), and other passive solid state dosimeters; diodes; metal oxide semiconductor field effect transistors (MOSFETs)‐based dosimeter, etc.^7^, ^13^, ^16^, ^17^, ^18^, ^19^, ^20^, ^21^, ^22^, ^23^, ^24^, ^25^, ^26^, ^27^, ^28^, ^29^, ^30^, ^31^, ^32^, ^33^, ^34^, ^35^, ^36^

The purpose of this work is to assess the skin doses in breast cancer treatments obtained with volumetric modulated arc therapy (VMAT) and three dimensional conformal radiation therapy (3DCRT) treatment techniques, using *in‐vivo* dosimetry, and compare the measured values with the doses calculated by the TPS.

## 2 MATERIALS AND METHODS

### 2.A MOSFET calibration

**Figure 1:**
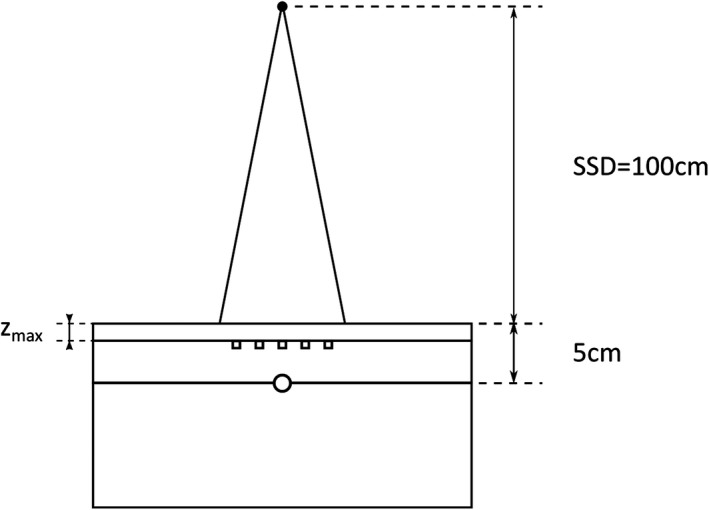
System calibration setup. The ionization chamber positioned at a depth of 5 cm and the MOSFET detectors at *Z*_max_ depth for the specific energy.

In this study, dual‐bias TN502RD MOSFET detectors fabricated by Thomson & Nielsen, Canada, were used. Before performing the skin dose measurements, the MOSFET detectors were calibrated using 4 and 6 MV photon beams using a Varian 2100C/D and a 2300IX linac (Varian Medical Systems, Palo Alto, USA), respectively. The detectors were calibrated using a PMMA MOSFET calibration jig (Arplay Medical Products #TN‐RD‐57‐30) in a solid water phantom (RW3 produced by PTW Freiburg GmbH, Freiburg, Germany) with source to surface distance (SSD) of 100 cm and a field size of 10 cm × 10 cm. A set of three detectors was placed in the central groves of the jig, with the flat side facing the beam, being the reference point at the position of the maximum dose (*Z*_max_) of each energy. A 0.6 cm^3^ Farmer ionization chamber (PTW 30013 PTW, Freiburg — Germany) was positioned at a depth of 5 cm for dose calibration verification (Fig. 1). The dose was calculated according to IAEA Technical Report Series (TRS) 398 protocol.^37^ The presence of the MOSFET did not disturb the ion‐chamber measurement as verified by our measurements. The MOSFET software (mobile MOSFET, Best^®^ medical Canada) enables the determination of the calibration factor (CF) by averaging three consecutive measurements which are user validated.

### 2.B MOSFET measurement system characterization methodology

**Figure 2:**
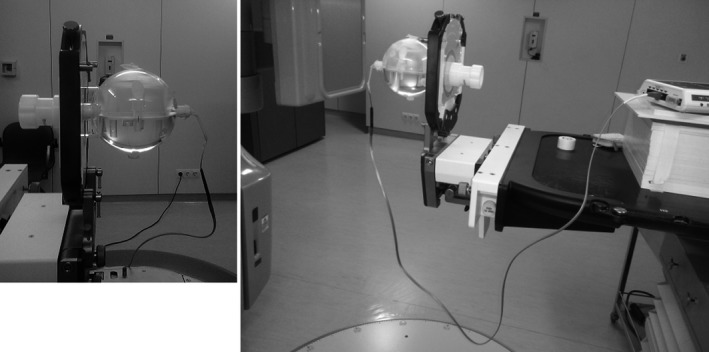
Angular dependence measurement setup, with Lucy 3D quality assurance phantom.

A thorough characterization of the MOSFET‐based dosimetry system was performed regarding linearity, reproducibility, and angular dependence for two photon energies commonly used in radiotherapy treatments of the breast (4 and 6 MV). All measurements, with the exception of the angular dependence, were performed using the standard setup above described for the calibration. The angular dependence was performed using a spherical phantom of 14 cm in diameter (Lucy 3D QA phantom – Standard Imaging, Middleton, USA) acquiring measurements with several beam incidences (45 degree increments; Fig. 2).

To assess the variation of the dosimeters response with dose, three MOSFET located at the build‐up position of each energy were irradiated in the range of 10 to 350 cGy with SSD = 100 cm. As a control measure (cross calibration), in the same procedure dose measurements were performed using the ionization chamber positioned at a 5 cm depth.

To assess the angular dependence, a MOSFET was placed in the center of the spherical phantom at the isocenter and a series of measurements was carried out by varying the rotation angle of the gantry. The MOSFET was positioned so that its flat side was facing the beam, with the gantry in the 0° position. The gantry angle varied between 0 and 315° in 45° increments.

### 2.C In‐phantom measurements methodology

**Figure 3:**
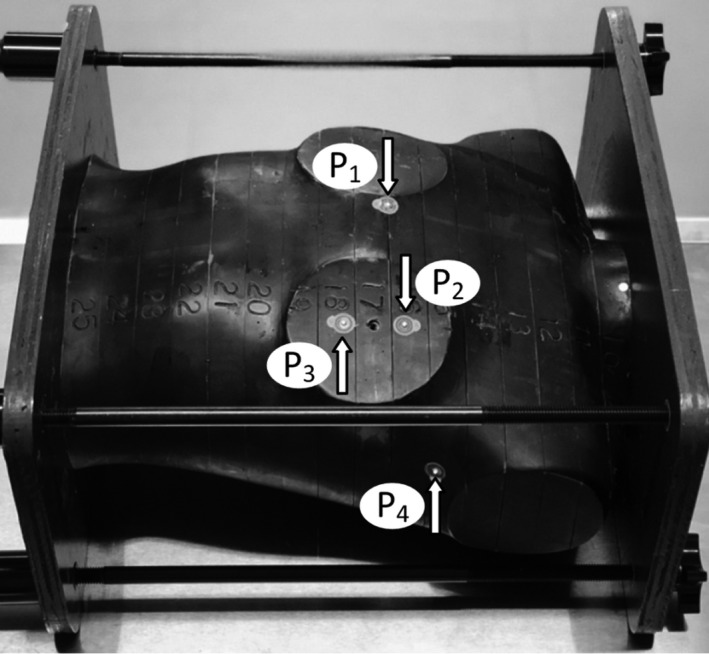
Position of the four measurement points on the female RANDO chest wall.

The *in‐phantom* measurements were performed using an anthropomorphic female RANDO phantom without breasts to simulate a post‐mastectomy patient. A CT scan of the phantom was acquired and four measurement points were selected on the phantom surface as seen in Fig. 3: two corresponding to the entrance and the exit of tangent beams and two over the planning target volume (PTV), corresponding to points 2 cm from each side of the usual location of the surgical scar.

The clinical importance of measuring dose in this area is due to the relative high probability of future relapse.^6^, ^7^, ^8^, ^9^ The fourth point (P_4_) was referenced on the axillar area, corresponding to the beam axis projection in the surface. The maximum sensitivity points of the MOSFET detectors were accurately placed on points P_1_–P_4_ and measurements were performed with and without bolus.

The output from the TPS calculation on these points was later compared with the MOSFET measured doses. For this purpose, we used the TPS version 13.5 Eclipse^®^ Varian (Varian Medical Systems, Inc., Palo Alto, CA, USA) using the Anisotropic Analytical Algorithm (AAA) with a calculation grid of 2.5 mm. 3DCRT treatment plans were performed in the RANDO phantom for the breast area, using two oblique opposing tangential fields in field‐in field technique with 4 MV photon beams. The VMAT plans were performed with 6 MV using two partial arcs, in the right side from 60° to 181° and in the left side from 340° to 179°.

A 3DCRT and a VMAT plan similar to a clinical case using 4 and 6 MV, respectively, was performed, with and without a bolus (SuperFlab, Eckert & Ziegler, 1 cm thick) for surface dose enhancement. The prescription was 2 Gy (average dose in the PTV) per fraction for a total dose of 50 Gy in 25 fractions. In the TPS, the bolus was added, based on previous acquires CT unit calibration and no extra CT was performed to the patient.

### 2.D *In‐vivo* measurements methodology

After characterization and dose measurements on the skin of the anthropomorphic phantom, *in‐vivo* measurements were performed in 20 patients with breast carcinoma undergoing treatment with two different irradiation techniques: 3DCRT and VMAT. For patients with high‐risk for skin involvement or inflammatory cancer, the hospital protocol recommends the use of bolus in the last 10 fractions for skin dose enhancement. From the total number of selected patients, fourteen underwent treatment with VMAT technique and six with 3DCRT technique.

**Figure 4:**
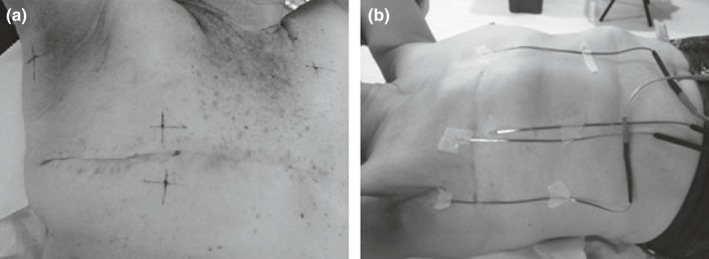
Patient skin measurement points tattooed during the computed tomography scan (a). Skin MOSFETS positioning on the patient prior treatment (b).

The CT scan was acquired with 2.5 mm slice spacing, extended from below the level of the orbits, when treatment includes regional lymph node irradiation or from the mandible, in the other cases, to about 5 cm below the ipsilateral or contralateral inframammary sulcus, in the case of mastectomized patients. During patient simulation, in addition to the positioning tattoos, four points corresponding to the measurement positions previously described (Section 2.C) were also tattooed [Fig. 4(a)].

A dose of 2 Gy per fraction for a total dose of 50 Gy was prescribed (PTV average dose) for all patients and both techniques according to the institutional protocol. Each plan was evaluated by analyzing PTV coverage and the dose‐volume histograms. The 3DCRT technique was performed using two oblique opposing tangential fields (with field‐in‐field technique) with 4 MV energy. The VMAT technique was performed with 6 MV using two partial arcs. Bolus (1 cm) was used to increase the skin dose in the last 10 fractions of the 25 fractions in both techniques.

For each patient, the MOSFET detectors were positioned on the respective tattoos and properly secured with adhesive tape [Fig. 4(b)]. The normal sequence of treatment was carried out and the dose values were registered for each point at the end of the treatment. These were compared with the values of the predicted dose on the TPS. The TPS calculations were performed at the depth of about 1 mm under the skin.

The *in‐vivo* measurements were performed for at least five fractions with bolus and five fractions without bolus, in order to increase the accuracy and reproducibility of the results. Each MOSFET was properly fixed on the initially referenced point on the patient skin for all measurements. After the end of the treatment, the MOSFET readings at each point were recorded. The measured dose for each position was then compared with the dose calculated by the TPS.

For each patient, the mean dose and the variability at each point were obtained. For a better interpretation of the results, the difference between the measured (*D*_M_) and the calculated dose by the TPS (*D*_TPS_) for each point was expressed as a percentage according to the Eq. (1):

(1) $$\nabla \%=\frac{D_{M}-D_{\mathrm{TPS}}}{D_{\mathrm{TPS}}}$$

## 3 RESULTS

### 3.A MOSFET measurement system characterization

#### 3.A.1 Linearity and reproducibility

**Figure 5:**
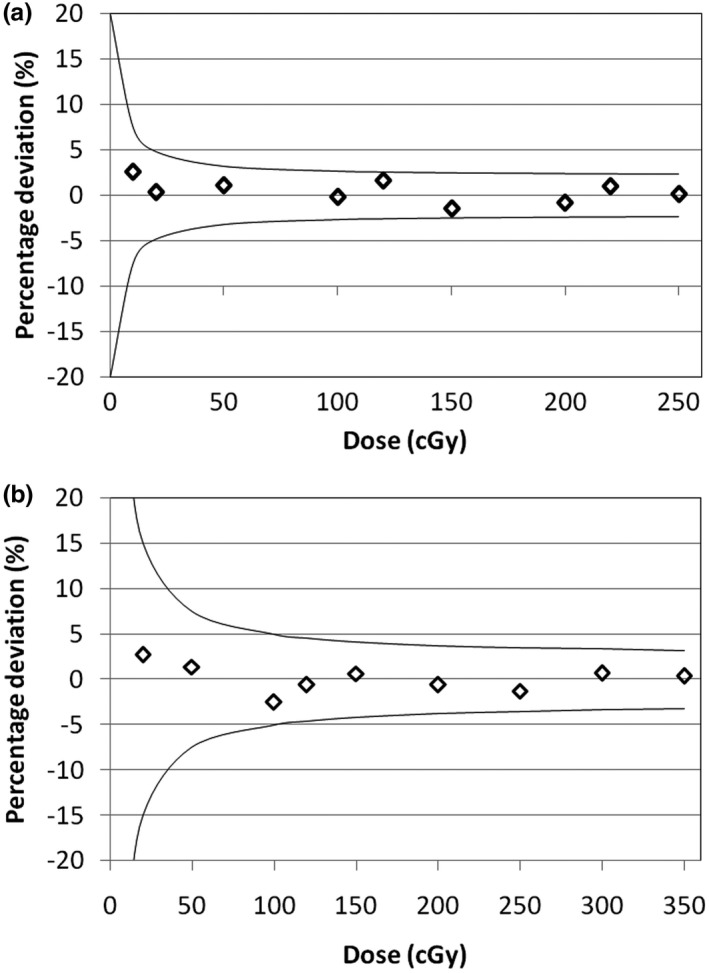
Linearity behavior of MOSFET detector for 4 MV (a) and 6 MV (b) energy.

Figure 5 represents the MOSFET dose dependence for 4 MV (a) and 6 MV (b) beams. The results were adjusted by a linear function *a x* + *b*, where *x* = number of monitor units (MU) and *a* and *b* are adjustable variables. The percent deviation of the measurement results from the fitting function can be seen in the graphs of Fig. 5. The uncertainty lines correspond to the 2*σ* value (95.4% confidence interval) extracted from different measurements for the same MU. It was found that the MOSFET system has a reproducible response, and shows good linear dependence in the dose range analyzed for both energies.

#### 3.A.2 Angular dependence

**Figure 6:**
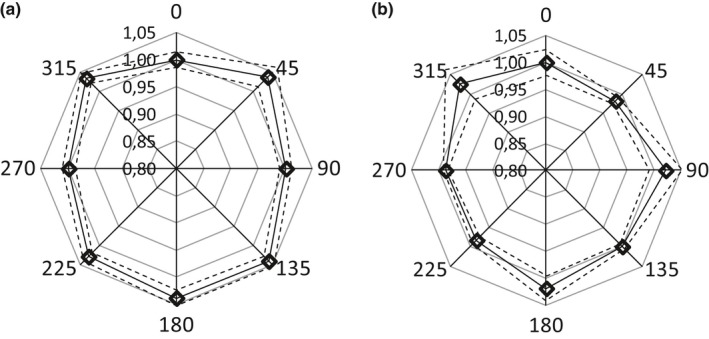
MOSFET angular response for 4 MV (a) and 6 MV (b).

The results are presented in the Fig. 6, for both studied energies. The standard deviations (%*σ*) obtained from all the considered directions were 1.86% and 1.67%, for 4 and 6 MV respectively, which are comparable within the ±2% variation over 360° as stated by the manufacturer.

### 3.B Phantom dose measurement

The results of the 3DCRT and VMAT plan irradiation of the female anthropomorphic phantom (*D*_M_) were compared with TPS calculated values (*D*_TPS_) with and without bolus.

**Figure 7:**
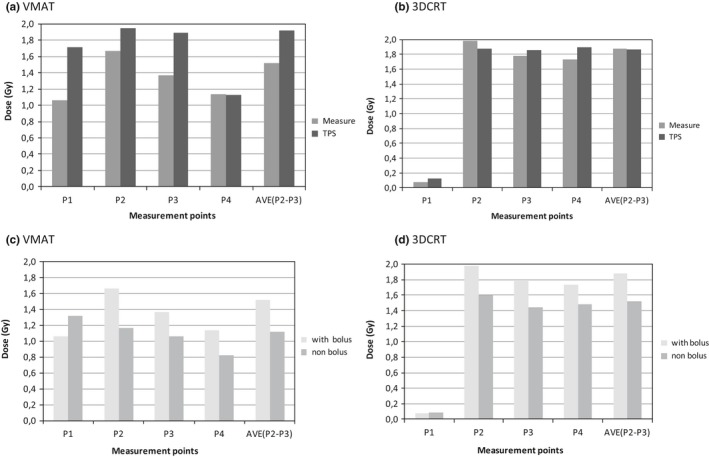
Difference between the measured and expected (TPS) dose, with bolus, at each point in the anthropomorphic phantom for volumetric modulated arc therapy (VMAT) (a) and three dimensional conformal radiation therapy (3DCRT) (b). Comparison of the measured dose with and without bolus for VMAT (c) and 3DCRT (d) technique. The locations of points P1–P4 are described in Section 2.C and shown in Fig. 3.

In Fig. 7 one can observe the difference between measured *vs*. calculated values as well as with *vs*. without bolus for both VMAT and 3DCRT.

As expected, partially due to the lower beam energy used, the measured surface dose presented higher values with 3DCRT (4 MV beam) technique compared with VMAT (6 MV beam) on the points above the PTV [Points P_2_ and P_3_ in Figs. 7(a) and 7(b)]. The anatomical upper inner quadrant of the contralateral breast (P_1_) showed higher dose to the surface with VMAT technique but within the locally accepted tolerance limit (less or equal to 20 Gy in total). In Figs. 7(c) and 7(d), one observes the surface dose difference at the surface of the phantom with and without bolus.

### 3.C Patient dose measurement

**Figure 8:**
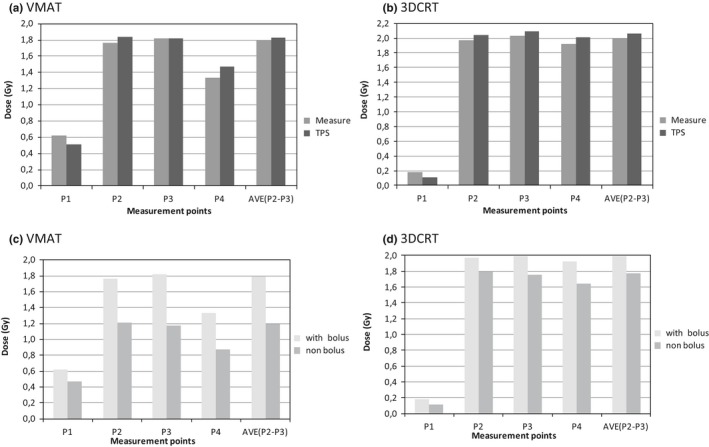
Average measured and expected (treatment planning system) doses with bolus at each point in patients for volumetric modulated arc therapy (VMAT) (a) and three dimensional conformal radiation therapy (3DCRT) (b). Comparison of the measured dose with and without bolus for VMAT (c) and 3DCRT (d) technique.

The obtained difference using the Eq. (1), ranged between 10% and 20% according to the considered points P_1_–P_4_ [Figs. 8(a) and 8(b)]. Analyzing the graphs in Figs. 8(c) and 8(d), the difference between the measured dose can be verified also “with bolus” vs “without bolus” for both 3DCRT and VMAT. In both cases, 80% of all measurements were within the range of ±20%, acceptance value for imprecision of the calculation of the dose by TPS recommended by the AAPM.^38^

## 4 DISCUSSION

### 4.A Phantom doses

In both VMAT and 3DCRT techniques, it is evident from Figs. 7(a) and 7(b) that, as expected, the surface dose with bolus is higher than to the surface dose without bolus. The obtained results agree with the literature, relating the increase of surface dose with the bolus presence on the irradiated area. The difference of measured and TPS calculated values are within the ±20%, corresponding to the accepted value for the imprecision of dose calculation recommended by the AAPM (20% in the buildup region).^38^

### 4.B Patient doses

In both VMAT and 3DCRT techniques, it is also evident from Figs. 8(a) and 8(b) that, as expected, the skin dose with bolus is superior to the skin dose without bolus. The obtained doses in all the points, except on P_1_, are consistent with the expected results. This might be due to the fact that, for patients, several different plans were used and the observed measured values are an average of several different geometries. Additionally, the effect of bolus is more evident in the VMAT than in the 3DCRT.

Comparing the skin dose at different points, at P_1_, anatomical upper inner quadrant of the contralateral breast, on average, the measured dose was lower than the dose calculated by the TPS for the 4 MV energy with the 3DCRT technique. This fact implies an overestimating of the dose in the P_1_. However, for the VMAT technique (6 MV) the TPS underestimates the dose at this point with an average difference of 22.8% in patients. At P_2_ and P_3_ the average measured doses agree with TPS calculation (<4%). In general, P_4_ is located in an area that presents large differences for both techniques (<10%).

Similar to the *in‐phantom* experiments, no correction factors were applied to the *in‐vivo* measurements. The same disparity trend between the measured and calculated values by the TPS was verified, compared to the results obtained in the phantom. Based on the analysis of the surface dose at the measurements points, it was verified that the points P_1_ and P_4_, corresponding to the anatomical upper inner quadrant of the contralateral breast and to the axillary area, present higher variability values, between calculated and measured, for the two treatment modalities, 3DCRT and VMAT. Since these points are located outside the area directly covered by the treatment fields, they are high dose gradient zones where the calculation is more imprecise. These are also subject to uncertainties of patient positioning and movement. In both techniques, the points on the PTV (P_2_ and P_3_), present higher homogeneity between calculated and measured doses. However, comparing the two treatment techniques, it was found that in the VMAT the TPS tends to underestimate the dose for these two points, contrary to the 3DCRT that tends to overestimate. These results are concordant in the two treatment phases with and without bolus. With the addition of the bolus in the final phase of treatment (after 15 treatment fractions without bolus), there was an increase of surface dose in the range of 5 to 20%, which is more significant in the VMAT technique. This variation from VMAT to 3DCRT can be compensated by changing the bolus thickness in the cases where an increase or a decrease of surface dose is desirable.

## 5 CONCLUSIONS

From the measurements made in the female anthropomorphic phantom RANDO, considering the treatment plan performed in the TPS, and without any correction factors, there was some discrepancy between the measured and the calculated values by the TPS, with more evidence for plan without bolus. However, this difference was within the ±20% error range, the value referred in the AAPM‐TG 53 for the TPS calculation imprecision in the buildup region. These measurements also demonstrate that the surface dose increased in the presence of the bolus when considering VMAT and 3DCRT. This increased surface dose is clearly higher in the VMAT technique. Since the treatment plan is very similar to that performed for treatment of patients with breast carcinoma, measurements using the RANDO phantom seem to indicate an easy and straightforward method of verifying surface dose (*in‐vivo*) applicable in actual clinical situations. It should be noted that this method may be used in patients with other pathologies, always taking into account the associated error.

## CONFLICT OF INTEREST

No conflicts of interest.
